# Novel modelling approaches to predict the role of antivirals in reducing influenza transmission

**DOI:** 10.1371/journal.pcbi.1010797

**Published:** 2023-01-06

**Authors:** Jason Asher, Annabelle Lemenuel-Diot, Matthew Clay, David P. Durham, Luis Mier-y-Teran-Romero, Carlos J. Arguello, Sebastien Jolivet, Diana Y. Wong, Klaus Kuhlbusch, Barry Clinch, Jean-Eric Charoin

**Affiliations:** 1 Leidos, Reston, Virginia, United States of America; 2 Roche Pharmaceutical Research and Early Development, Pharmaceutical Sciences, Roche Innovation Center, F. Hoffmann-La Roche Ltd, Basel, Switzerland; 3 Office of the Assistant Secretary for Preparedness and Response, U.S Department of Health and Human Services, Washington, District of Columbia, United States of America; 4 F. Hoffmann-La Roche Ltd, Basel, Switzerland; 5 Roche Products Ltd., Welwyn Garden City, United Kingdom; University of Washington, UNITED STATES

## Abstract

To aid understanding of the effect of antiviral treatment on population-level influenza transmission, we used a novel pharmacokinetic–viral kinetic transmission model to test the correlation between nasal viral load and infectiousness, and to evaluate the impact that timing of treatment with the antivirals oseltamivir or baloxavir has on influenza transmission. The model was run under three candidate profiles whereby infectiousness was assumed to be proportional to viral titer on a natural-scale, log-scale, or dose–response model. Viral kinetic profiles in the presence and absence of antiviral treatment were compared for each individual (N = 1000 simulated individuals); subsequently, viral transmission mitigation was calculated. The predicted transmission mitigation was greater with earlier administration of antiviral treatment, and with baloxavir versus oseltamivir. When treatment was initiated 12–24 hours post symptom onset, the predicted transmission mitigation was 39.9–56.4% for baloxavir and 26.6–38.3% for oseltamivir depending on the infectiousness profile. When treatment was initiated 36–48 hours post symptom onset, the predicted transmission mitigation decreased to 0.8–28.3% for baloxavir and 0.8–19.9% for oseltamivir. Model estimates were compared with clinical data from the BLOCKSTONE post-exposure prophylaxis study, which indicated the log-scale model for infectiousness best fit the observed data and that baloxavir affords greater reductions in secondary case rates compared with neuraminidase inhibitors. These findings suggest a role for baloxavir and oseltamivir in reducing influenza transmission when treatment is initiated within 48 hours of symptom onset in the index patient.

## Author summary

The relationship between the amount of influenza virus shed when a person is infected and how infectious that person is as a result is yet to be established. Understanding this relationship is important for controlling the spread of influenza virus in a population. Here, we tested three models that describe this relationship to investigate how the timing and type of antiviral treatment would be expected to affect the transmission of influenza virus. We compared our simulated data with those from a clinical trial of an antiviral (baloxavir marboxil; BLOCKSTONE post-exposure prophylaxis study) to identify which of the three models best describes the relationship between influenza viral shedding and the infectiousness of a person. We found that two of the three models accurately describe this relationship, and that administration of antiviral treatment within 48 hours of symptom onset would be expected to reduce onward influenza transmission.

## Introduction

Seasonal influenza virus infections cause significant global morbidity and mortality annually, despite the availability of vaccines [1]. In the United States, the Centers for Disease Control and Prevention estimated that between 2010 and 2020, 9–41 million people were ill with influenza annually, 140,000–710,000 were hospitalised, and 12,000–52,000 died [2].

Oral antivirals such as oseltamivir and baloxavir marboxil (hereafter referred to as baloxavir) can be effective treatments for influenza, particularly when administered shortly after symptom onset, and are also beneficial in prophylactic settings [3–7]. Oseltamivir is a neuraminidase inhibitor (NAI) given as twice-daily doses for five days to treat influenza [8]. It acts late in the virus replication cycle, restricting the release of progeny virions from infected cells [8,9]. By contrast, baloxavir is a cap-dependent endonuclease inhibitor given as a single dose, which acts earlier in the replication process to arrest transcription of viral mRNA [10,11]. Antiviral treatment leads to a reduction in viral titer and shedding in infected patients; recent studies have shown that baloxavir induces a more rapid reduction in viral titer than oseltamivir across all populations examined [3,5,7,12,13]. In the Phase 3 BLOCKSTONE study (EudraCT: 2020-000696-20), single-dose baloxavir exhibited significant post-exposure prophylactic efficacy versus placebo in household contacts of patients with influenza [14]. In total, 752 household contacts of 545 index patients were randomized to receive baloxavir or placebo [14]. As BLOCKSTONE was conducted in Japan where treating influenza with antivirals is standard of care, all index patients were treated with antivirals; 52.7% received baloxavir, 31.4% oseltamivir, and 16.0% another NAI. In this study, index patients predominantly had influenza A virus infection (95.6%) [14].

An assumption is often made in the modelling literature that a correlation exists between viral load and patients’ infectiousness; however, the nature of this correlation remains unclear [15–20]. For example, an individual-based simulation model of influenza epidemics assumed that an infected individual remained infectious for six days, with infectiousness proportional to their viral load [16], whereas a different study assumed a logarithmic relationship between viral load and infectiousness [21]. Other studies have debated the importance of the effects of large droplets versus small aerosol particles on infectiousness, and challenge the correlation between nasal viral load and exhaled virus [17–19]. Furthermore, the anatomical sampling site may also affect the resultant viral load and, by extension, the predicted infectiousness.

Here, we present a novel modelling approach to test the correlation between viral load and infectiousness and evaluate how oseltamivir and baloxavir might impact influenza transmission at the population level via an effect on viral load. This involved developing a viral dynamic model (herein referred to as the pharmacokinetic [PK]–viral kinetic [VK] model) to describe the effect of different treatments on the viral load time course. Subsequently, three different infectiousness profiles employing unique functions were developed to simulate transmission dynamics (i.e., secondary case reduction) in different scenarios, including investigations of the impact of timing of treatment initiation. Results were compared with clinical trial data from BLOCKSTONE.

## Results

### PK–VK model

**Fig 1:**
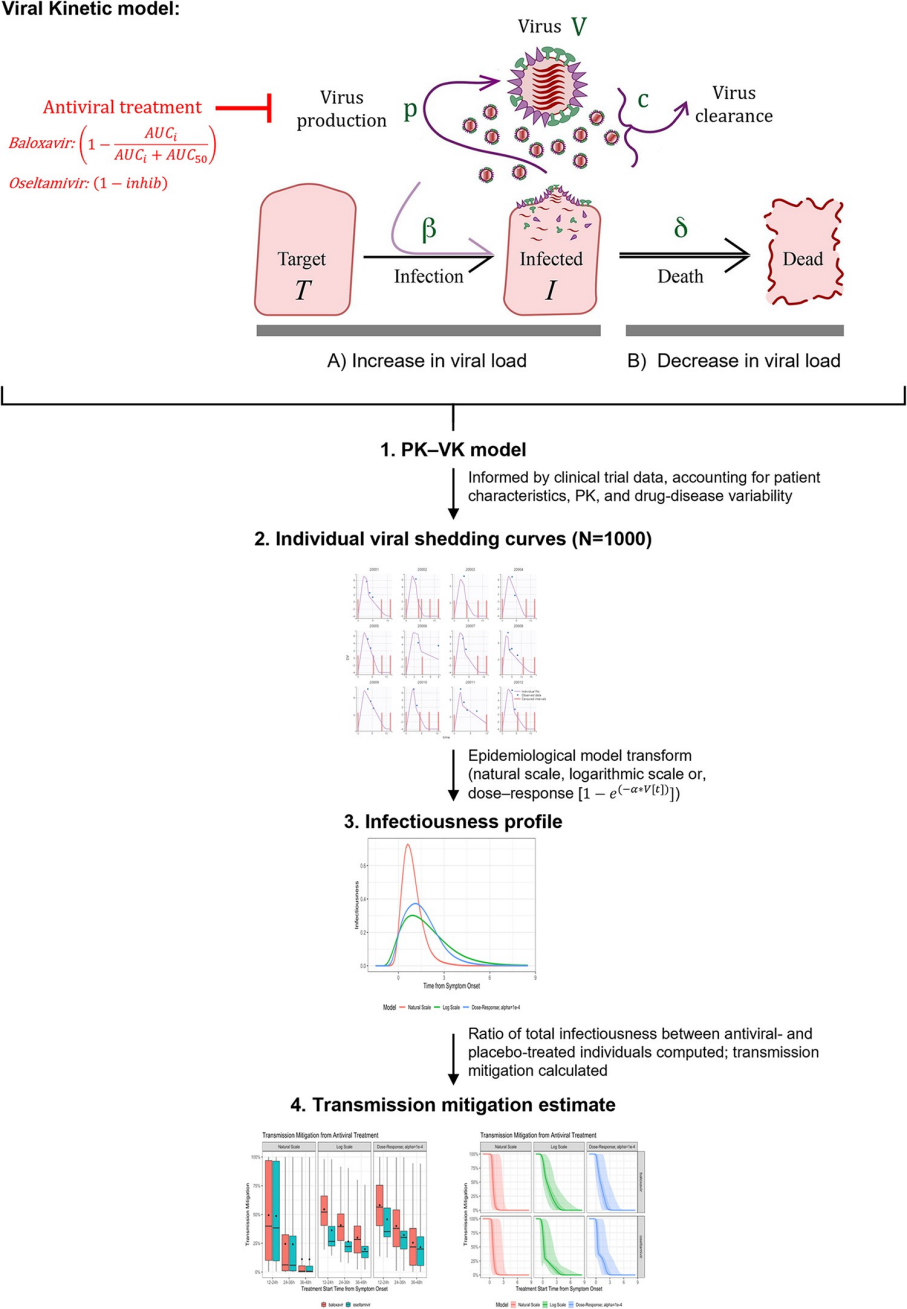
Schematic representation of viral kinetic model and study overview. The viral kinetic model assumes that the initial viral load increases (A) by infection (rate β) of and replication within target cells (T), which subsequently shed progeny virions (free virus, V; virus production rate, p). Antiviral treatment prevents the increase of viral load via inhibition of virus production; for baloxavir, this was informed by PK estimates from clinical trial data, whereas an inhibition factor was used for oseltamivir (in lieu of PK model data). The viral load decreases (B) with the death (rate δ) of infected cells (I) and by clearance of free virus (rate c). The PK–VK model was used to generate viral shedding curves (i.e. viral titer time courses) for 1000 simulated individuals treated with either baloxavir, oseltamivir, or placebo. These shedding curves were transformed to generate infectiousness profiles using one of three epidemiological models, whereby infectiousness was assumed to be proportional to viral load on natural-scale, a logarithmic-scale, or a dose–response transform. Transmission mitigation (i.e. the reduction in secondary transmission) was computed by comparing the area under the respective model-transformed infectiousness profiles for individuals treated with an antiviral or placebo. Figure adapted from Kamal et al. 2015 [23]. PK, pharmacokinetics; VK, viral kinetics.

**Fig 2:**
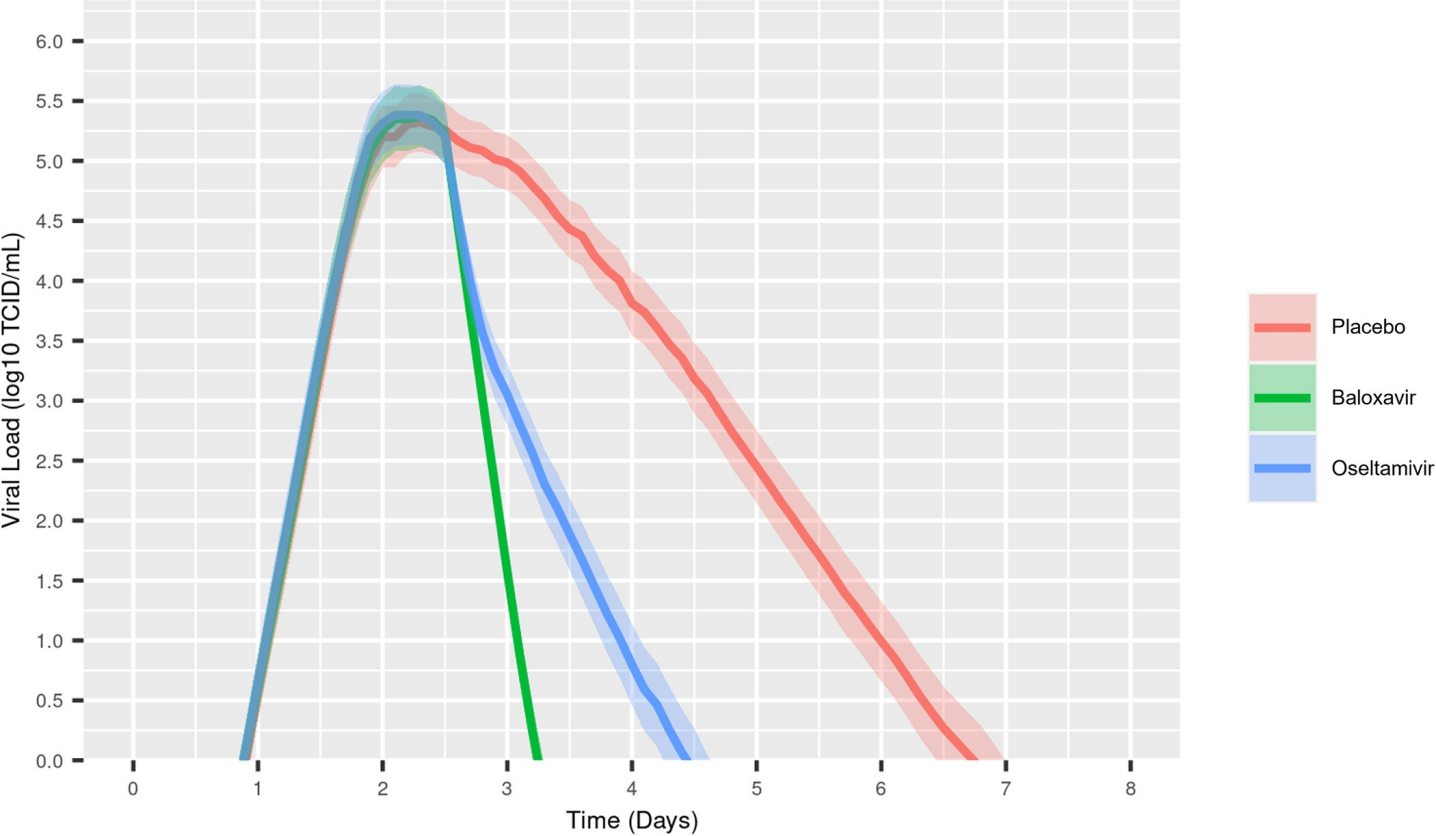
Influenza virus titer time course in response to antiviral treatment, simulated using the pharmacokinetic–viral kinetic model. The line represents the median of 1000 simulated individuals with the shaded areas corresponding to the 95% confidence interval around this median. Symptoms were assumed to start 36 hours after infection, and treatment was administered 24 hours after symptom onset (sampled from the observed distribution).

A schematic representation of this study is presented in Fig 1. A PK–VK model was developed as an ordinary differential equation system model, which was subsequently used to generate viral titer time-courses for 1000 simulated individuals treated with either baloxavir, oseltamivir, or placebo. Disease- and drug-related parameters of the within-host PK–VK model were estimated by fitting the viral titer in log-scale, using a stochastic approximation of expectation–maximization (SAEM) algorithm as developed into the Monolix software [22] using data from Phase II and III studies of baloxavir in adult patients with influenza (T0821 [JapicCTI-153090]; T0831 [NCT02954354]; T0832 [NCT02949011]; S1 Table) [3,5]. This model successfully described the data (S1 and S2 Figs); therefore, there was no need to develop a more complex model. The model parameters from this mixed–effect model were well estimated using some between–subject variability on V0, beta, and p, as well as an additive error model for the residual. These model parameters are displayed in Table 1. Individual PK exposures, measured as the area under the PK time-course profile (AUC), were the best driver of the individual antiviral effect, via inhibition of virus production. A low AUC_50_ (AUC at 50% of the inhibition effect) indicated a substantial effect with low antiviral exposure. There were no observed differences between otherwise-healthy and high-risk patients for any disease or drug parameters. Subsequently, simulations were performed to generate 1000 individual viral titer time courses in a Caucasian population infected with influenza type A for each treatment group, as this population is the one accounted for in the model development. Individual viral titer time-course profiles for patients treated with placebo, baloxavir, or oseltamivir, simulated using the PK–VK model, were averaged (median of 1000 simulated individuals with 95% confidence intervals) and are shown in Fig 2. Compared with placebo, antiviral reduces the overall duration of infectiousness by reducing the time to cessation of viral shedding; baloxavir induces a more rapid reduction in viral load compared with oseltamivir.

**Table 1:** Parameter estimates of the within-host pharmacokinetic–viral kinetic model.

Parameter	Definition	Units	Estimate	%SEM
**System**				
V0	Log_10_ (amount of virus at t = 0)	Log_10_(TCID_50_/mL)	–3.42	2
β	Target cell infection rate	(TCID_50_/mL)^-1^∙ days^-1^	1.81∙10^−6^	1
p	Virus production rate	(TCID_50_/mL) ∙ days^-1^	0.661	1
c	Virus clearance rate	days^-1^	14.8	3
δ	Infected cell clearance rate	days^-1^	7.07	3
**Drug**				
AUC_50_	Drug sensitivity on p inhibition	ng.h/mL	0.434	23
**Random Effect**				
Ω_V0_	Inter-individual variability of V0	CV%	27.8	23
Ω_β_	Inter-individual variability of β	CV%	28.4	9
Ω_δ_	Inter-individual variability of δ	CV%	64.7	4

AUC_50_, area under the concentration-time curve; CV, coefficient of variation; h, hours; SEM, standard error of the mean; TCID, median tissue culture infectious dose.

### Epidemiological models

**Fig 3:**
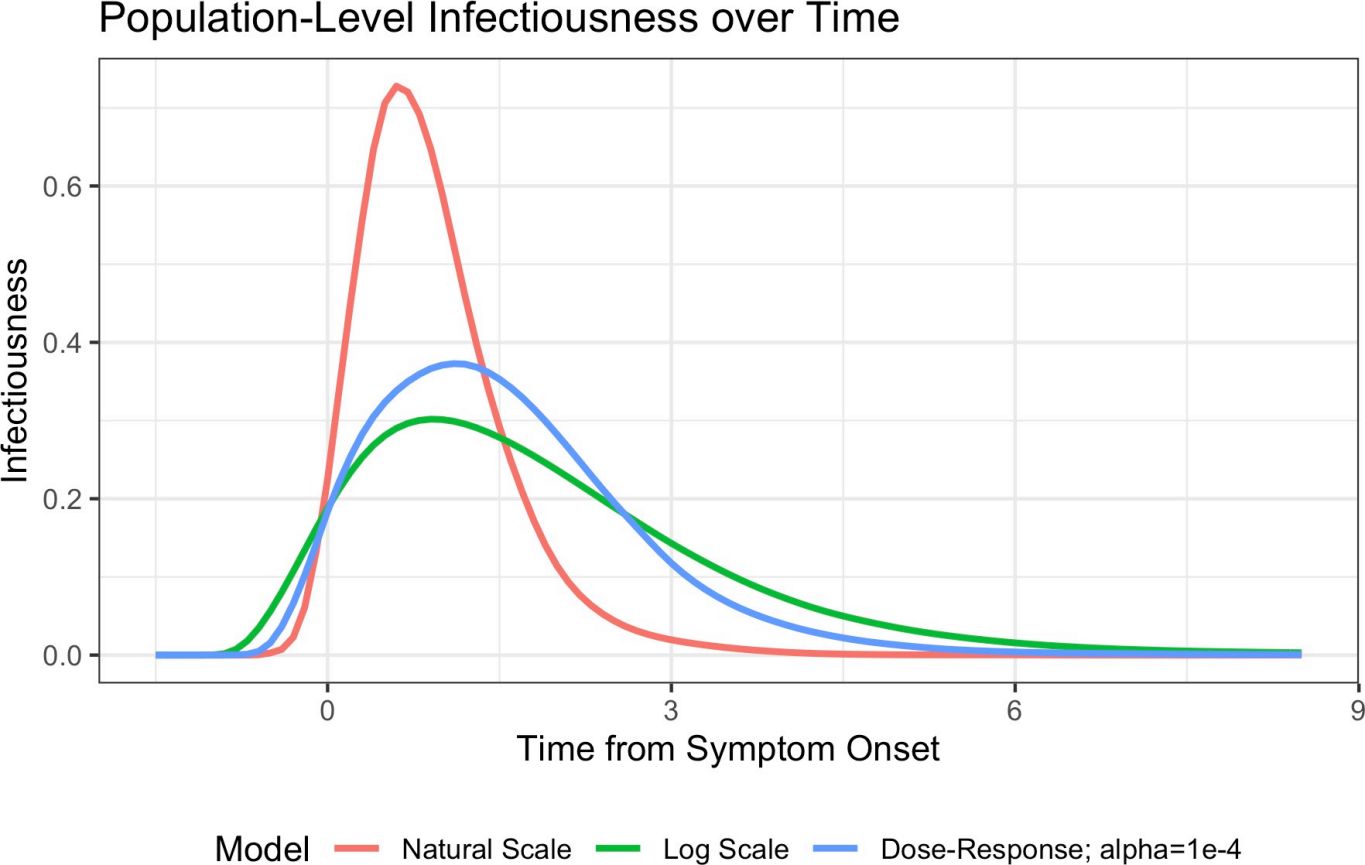
Estimated population-level influenza infectivity profiles over time from symptom onset according to the three epidemiological models studied. The probability densities are shown for the timing of secondary infections expected under each of the models considered. The VK model was used to simulate individual viral shedding trajectories and the epidemiological model was used to translate these into infectiousness profiles over time. These individual profiles were then normalized so that the area under the curve was equal to 1 (once averaged), with each individual contributing to the average proportional to their time-integrated total infectiousness.

Viral titer time-course profiles were subsequently transformed into infectiousness profiles for each simulated individual using one of three epidemiological models (natural-scale, log-scale, or dose–response). Infectiousness profiles generated using the epidemiological models, and describing population infectivity as a function of time, are shown in Fig 3. Of the three models considered, the natural-scale model estimated a sharp increase in infectivity concurrent with symptom onset that attenuated shortly thereafter and resulted in no infectivity from approximately four days after symptom onset. By contrast, the dose–response and log-scale models predicted lower peak infectiousness but longer durations of infectivity, achieving resolution approximately five and seven days after symptom onset, respectively (Fig 3).

### Timing of treatment initiation after symptom onset affects the mitigation potential of antivirals

**Fig 4:**
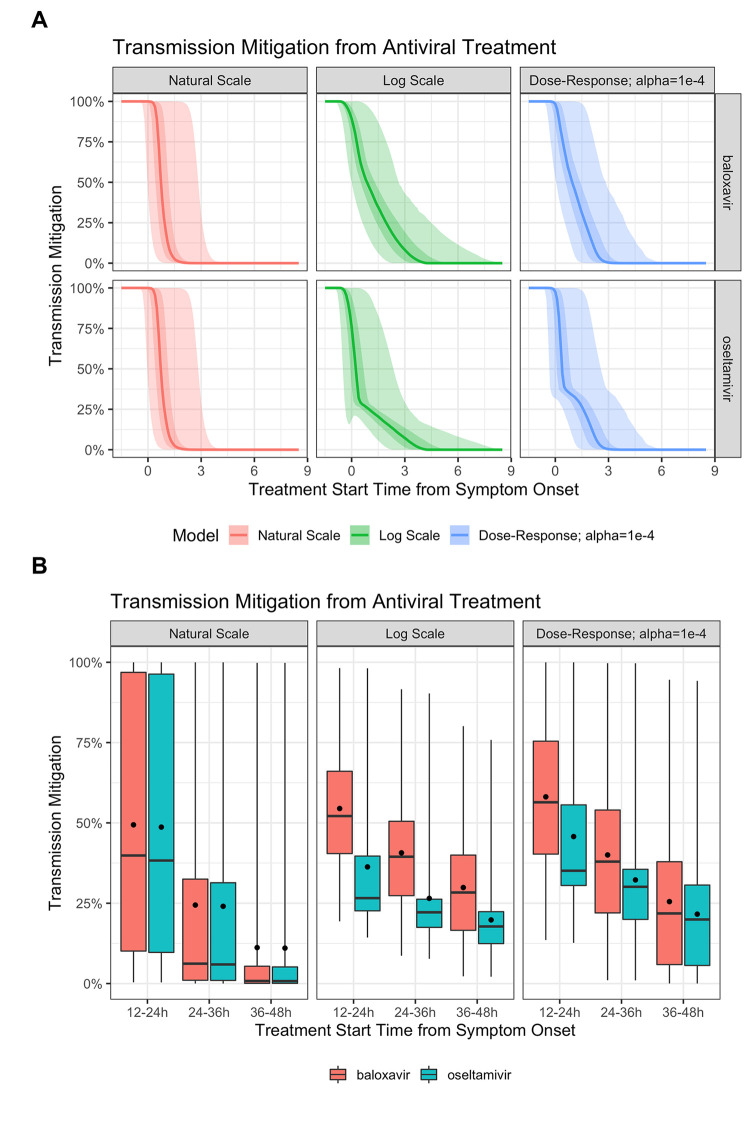
Estimated mitigation of influenza transmission by baloxavir and oseltamivir. Estimated mitigation of influenza transmission by baloxavir and oseltamivir according to treatment initiation time from symptom onset using natural-scale, log-scale, and dose–response models, with time presented on a continuous scale (A) or as discrete time intervals (B). Both figures show interindividual variability. (A) The line represents the median; lighter shaded areas correspond to the 95% confidence interval around this median, and darker shaded areas show the interquartile range. (B) The boxplots show median, interquartile range, and 95% confidence intervals; the dots correspond to the mean.

Viral transmission mitigation was calculated from the infectiousness profiles by computing the ratio of an individual’s total infectiousness in the presence and absence of antiviral treatment at different initiation time points. The effects of antiviral treatment with baloxavir or oseltamivir on influenza transmissibility over time, estimated using natural-scale, log-scale, and dose–response models, are shown in Fig 4. There was substantial inter-person variation in infectiousness as well as response to drug mitigation and thus transmission mitigation (i.e., reduction in expected transmission over the course of infection for a treated individual relative to their untreated cumulative infectiousness) potential over time. When transmission mitigation equals 100%, there is no secondary transmission; at 50%, the total time-integrated expected number of secondary infections from an individual is halved; and at 0% there is no change relative to the untreated case. The natural-scale model predicted a very rapid attenuation of transmission potential with antiviral treatment (Fig 4A). Compared with no treatment, 50% and 0% transmission mitigation were achieved when treatment with either baloxavir or oseltamivir was initiated in the median person 1 day and 12–24 hours after symptom onset, respectively. In contrast, the log-scale model predicted that later treatment would offer some degree of transmission reduction (Fig 4A). With baloxavir, 50% and 0% transmission mitigation were achieved when treatment was initiated in the median person approximately 1 and 4 days after symptom onset, respectively. With oseltamivir, 50% and 0% transmission mitigation were achieved upon initiating treatment <1 day and 4 days following symptom onset, respectively. The dose–response model estimated an intermediate effect of antiviral treatment timing on transmission mitigation potential (Fig 4A); 50% and 0% transmission mitigation were achieved in the median person when treatment with baloxavir was initiated <0.5 day and 3 days post symptom onset, respectively. With oseltamivir, 50% and 0% transmission mitigation were achieved 1 day and 3 days after symptom onset, respectively.

The difference between the transmission mitigation potential of baloxavir and oseltamivir was more pronounced with early treatment (Fig 4B). The natural-scale model predicted similar transmission mitigation potentials for oseltamivir and baloxavir across time periods–when transmission mitigation profiles were generated using discrete time periods of treatment initiation after the start of symptom onset (i.e., 12–24, 24–36, and 36–48 hours)–with only very early treatment (12–24 hours) impacting transmission. Using the natural-scale model, transmission mitigation was 39.9%, 6.2%, and 0.8% for baloxavir and 38.3%, 5.9%, and 0.8% for oseltamivir when treatment was initiated 12–24, 24–36, and 36–48 hours post symptom onset, respectively. However, for both the log-scale and dose–response models, the difference between the transmission mitigation potential of baloxavir and oseltamivir was more pronounced following treatment initiation at 12–24 and 24–36 hours after symptom onset. Using the log-scale model, the predicted transmission mitigation was 52.1%, 39.5%, and 28.3% for baloxavir and 26.6%, 22.2%, and 17.8% for oseltamivir when treatment was initiated 12–24, 24–36, and 36–48 hours post symptom onset, respectively. Whereas using the dose–response model, the predicted transmission mitigation was 56.4%, 38.0%, and 21.8% for baloxavir, and 35.1, 30.1%, and 19.9% for oseltamivir when treatment was initiated 12–24, 24–36, and 36–48 hours post symptom onset, respectively.

### Baloxavir is predicted to reduce secondary case rate among household contacts

**Fig 5:**
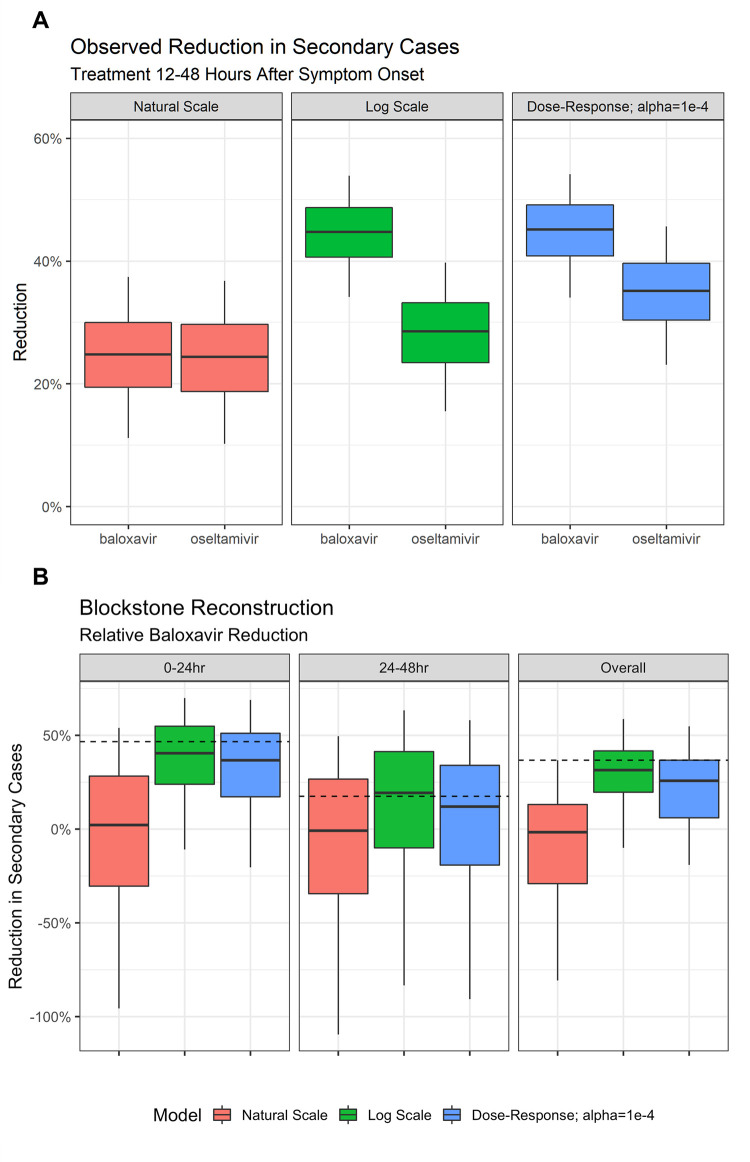
Estimated reduction in secondary cases of influenza with baloxavir and oseltamivir. The boxplots show median, interquartile range, and 95% confidence intervals. (A) Estimated reduction in secondary case rate among household contacts with prophylactic baloxavir and oseltamivir according to natural-scale, log-scale, and dose–response models, assuming treatment within 12–48 hours of symptom onset in the index case. Variability across individuals is shown. (B) Relative reduction in secondary cases with baloxavir treatment, comparing simulations with observed clinical trial data. Data are shown for baloxavir treatment at 0–24 and 24–48 hours after symptom onset in the index case, as well as overall (irrespective of treatment initiation time). The dashed line indicates data from the Phase 3 BLOCKSTONE study [14]. Variability in the observed reduction in secondary cases across simulations of the clinical trial is shown, which includes sampling and individual variation effects.

The epidemiological models (natural-scale, log-scale, or dose–response) were used to estimate the reduction in secondary case rate among household contacts, assuming the index patient was treated with either baloxavir or oseltamivir within 12–48 hours of symptom onset. In a household transmission trial setting, all three models predicted a reduction in secondary case rate following treatment of the index case with oseltamivir or baloxavir 12–48 hours after symptom onset (Fig 5A). Overall, 10,000 simulations of the trial were conducted with ~480 patients per arm, including drop-out. The natural-scale model placed the greatest weight on transmission near the time of symptom onset, and thus predicted the lowest rate of mitigation of 24.8% and 24.3% for baloxavir and oseltamivir, respectively. The log-scale and dose–response models predicted higher rates of transmission mitigation with the use of antivirals; baloxavir induced a greater reduction in secondary case rate compared with oseltamivir in both models. Using the log-scale and dose–response models, respectively, the predicted reduction of secondary cases was 44.8% and 45.1% for baloxavir, and 28.5% and 35.1% for oseltamivir.

Simulated data were compared with those obtained in the Phase 3 BLOCKSTONE study (Fig 5B and Table 2) for baloxavir, relative to an NAI [14]. It was assumed that the NAI was oseltamivir, as most of the index patients who did not receive baloxavir in BLOCKSTONE received oseltamivir (52.7% of index patients received baloxavir, 31.4% oseltamivir and 16.0% another NAI) [14]. Observed data from BLOCKSTONE revealed an overall 36.7% reduction in secondary cases with baloxavir treatment relative to a non-baloxavir comparator (S2 Table). The reduction in secondary cases reached 46.3% when treatment was initiated within the first 24 hours after symptom onset, falling to 17.6% if treatment began 24–48 hours after symptom onset (S2 Table). Of the three epidemiological models, the log-scale model most closely aligned with the observed BLOCKSTONE data (Table 2). Using the log-scale model, compared with NAI treatment, an overall 31.5% reduction in secondary cases was predicted with baloxavir; if treatment of the index case was initiated within 24 hours of symptom onset, secondary case reduction was 40.5%, and was 19.3% if treatment began 24–48 hours after symptom onset. The dose–response model underestimated the magnitude in secondary case rate reduction with baloxavir compared with the observed BLOCKSTONE data; however, the decrease in secondary case rate reduction with later treatment initiation times was depicted. The natural-scale model failed to accurately reconstruct the observed data.

**Table 2:** Observed and simulated transmission mitigation following treatment of index patients with baloxavir relative to NAI treatment in the BLOCKSTONE study.

Timing of treatment initiation after symptom onset (index patient)	Percentage change with baloxavir (relative to NAI)	Interquartile range
**BLOCKSTONE (observed)**		
Overall	36.7	–
0–<24 hours	46.3	–
24–48 hours	17.6	–
**Natural-scale model**		
Overall	–1.6	–29.1, 13.1
0–<24 hours	2.2	–30.4, 28.3
24–48 hours	–0.8	–34.4, 26.7
**Log-scale model**		
Overall	31.5	19.7, 41.7
0–<24 hours	40.5	23.9, 54.9
24–48 hours	19.3	–10.0, 41.3
**Dose–response model**		
Overall	25.8	6.0, 36.8
0–<24 hours	36.7	17.2, 51.1
24–48 hours	12.0	–19.2, 34

NAI, neuraminidase inhibitor

## Discussion

The novel modelling approaches adopted in this study predict a role for baloxavir and oseltamivir in reducing influenza transmission, and highlight that treatment with baloxavir in index patients may confer greater reductions in secondary case rates than NAIs. Furthermore, the results illustrate how these methods can help increase our understanding of how antivirals impact influenza infectiousness. Comparisons of population-level infectiousness simulations with the published literature suggest that the dose–response model of infectiousness compares favourably with prior estimates, whereas the natural-scale model underestimates the duration of infectiousness [20,21]. Additionally, an initial comparison of treatment-induced transmission mitigation simulations with clinical data from the BLOCKSTONE study [14] suggests that the log-scale model of infectiousness is the most consistent with observed data, closely followed by the dose–response model. However, such comparisons may have some limitations due to the differing ethnicities of simulated patients (Caucasian) and those enrolled in BLOCKSTONE (Japanese). Ethnic sensitivity analyses and future clinical trials of influenza transmission will aid the refinement of this model.

While all three models (natural-scale, log-scale, and dose–response) take into account the actual amount of virus shed by an infected individual, the probability that each virion transmits to and replicates in an infected contact varies, subsequently altering infection time-course dynamics. The relationship between the timing of treatment initiation and the mitigation of onward transmission in these models is complex, with apparent biphasic and tail non-monotonic patterns. According to the natural-scale model, the likelihood of individual virions causing infection is very low, and thus risk of infection is greatest when viral shedding is greatest (at symptom onset) but very low by 3 days post-symptom onset. According to the log-scale and dose–response models, individuals that shed virus at moderate levels for longer durations still contribute substantially to the risk of infection at later time points after symptom onset. As these individuals have lower rates of viral clearance, oseltamivir treatment (which modulates clearance directly) can have an effect more comparable to that of baloxavir at these later time points. Relatedly, for a very small proportion of modelled individuals, early treatment could result in more moderate but also more prolonged viral shedding compared with slightly later treatment, and thus according to the dose–response model could result in slightly increased overall transmission.

Although this model is mechanistically derived, it assumes that viral shedding from the upper respiratory tract is functionally related to infectiousness. However, some studies suggest there is no strong correlation between nasal viral load and onward transmission [17–19]. The origin (upper versus lower respiratory tract) and size of respiratory secretions (large droplets versus aerosols) potentially contribute to this lack of correlation [17,18]. For example, the authors of one study suggested that smaller aerosol droplets, which have an abundance of viral RNA and are thought to play an important role in viral transmission, might be generated in the lower respiratory tract [18]. Furthermore, the authors proposed that the viral load of the lower respiratory tract may differ from that of the upper respiratory tract [18]. As such, this may have implications for our model design. Studies elucidating the relationship between viral shedding, transmission modalities, and relative infectivity are required to refine epidemiological modelling and better inform containment/mitigation strategies. Future modelling efforts can also be performed to evaluate alternate infection models. As additional data become available, ideally across a range of viral loads, greater clarity can hopefully be gained to determine the most accurate functional relationship between viral load and infectiousness.

The model requires further enhancement and interrogation. Future studies will challenge the model with existing results from the literature regarding the effect of oseltamivir on influenza transmission, and will tailor the model to account for differences in age, based on the inherently unique viral kinetic profiles of paediatric versus adult populations. In addition, the model will be extended to include Asian populations and patients with influenza B infection. Results from future clinical studies will be used to identify which of the three epidemiological models most accurately depicts the relationship between viral kinetics and infectiousness.

These findings indicate that antivirals impact the infectiousness of index patients when administered within 48 hours of symptom onset, and may reduce secondary case rates during influenza outbreaks in households and other close-contact settings. Our data suggest that the greatest reductions in secondary case rate occur when antiviral treatment is administered 12–24 hours post symptom onset, and that baloxavir may afford greater reductions than NAIs. This highlights the need for early identification of influenza symptoms, rapid diagnosis, and the subsequent prompt administration of effective antiviral therapy to reduce onward transmission. Further modelling studies and clinical trial data are required to determine the impact of antiviral treatment on influenza transmission outside of household environments and during pandemic situations.

## Methods

### Overview

Firstly, the PK–VK model was developed to understand the population-level variation in viral shedding trajectories over time, and in response to treatment with baloxavir, oseltamivir, or placebo; viral shedding curves were generated for 1000 simulated individuals. These viral shedding curves were translated into infectiousness profiles for each simulated individual using one of three epidemiological models which assumed a functional relationship between nasal viral shedding and infectiousness. For each individual, the ratio of their total infectiousness with and without antiviral treatment was computed; subsequently, viral transmission mitigation was calculated. The impact of timing of antiviral treatment initiation was also investigated. Results were compared with clinical trial data from a post-exposure prophylaxis study of baloxavir, BLOCKSTONE.

### Pharmacokinetic (PK)–viral kinetic (VK) model

The PK–VK model was developed with Monolix software using data from Phase II and III studies of baloxavir in adult patients with influenza (T0821 [JapicCTI-153090]; T0831 [NCT02954354]; T0832 [NCT02949011]) [3,5,11] where individual viral titer time courses were available following treatment administration. An overview of the included populations and treatment initiation times are provided in S1 Table. With regards to this specific analysis where the objective was to describe the effect of baloxavir on reducing influenza transmission in the US population, only data from Caucasian populations infected with influenza A for each treatment group were included in these analyses. The within-host VK model was based on a classic VK modelling approach (Fig 1) and used the following differential equations [23,24]:

$$\frac{dT}{dt}=-\beta TV$$ (1)

$$\frac{dI}{dt}=\beta TV-\delta I$$ (2)

$$\frac{dV}{dt}=pI-cV$$ (3)

Variables and parameters are defined in Table 1. With regards to baloxavir, the effect of antiviral treatment was characterized as an inhibition of the production rate *p* using an inhibition factor equal to:

$$\left(1-\frac{{AUC}_{i}}{{AUC}_{i}+{AUC}_{50}}\right)$$

with *AUC*_*i*_ (ng.h/mL) defined as the area under the PK time-course profile over the dosing interval, derived for each individual from the population PK model, and AUC_50_ (the AUC at 50% of the inhibition effect). With regards to oseltamivir, the effect was characterized by an inhibition of the production rate using a factor of inhibition, as PK data and a model were not available. The estimation process assumed that: infection occurred at time (t) = 0 days with an estimated individual viral titer baseline (V0); the initial number of target cells and infected cells similar for all individuals and equal respectively to 4.10^8 and 0.000001; symptom onset started at t = 1.5 days after infection; and treatment started at t = 1.5 days + time from symptom onset (TOS), where TOS was individually reported into the clinical trials’ database (not publicly available). The model building was performed using the classical steps of the mixed effect modelling: determination of the structural model, assessment of the between subject variability on the different parameters, potential demographic covariate selection, and structure of the error model. The model selection was performed based on the goodness-of-fit plot, accuracy of the parameter estimation, and log-likelihood maximization.

Once the model parameters related to the disease characterisation and drug effect were estimated, the PK–VK model was used to simulate viral titer time courses per individual, in response to different treatments (placebo, oseltamivir, or baloxavir). These individual simulated viral titer time courses were used to inform the epidemiological models.

For each treatment group, simulations (*i*) of 1000 patients were performed using Berkeley Madonna and R (v4.0) software. In order to calibrate the epidemiological models, simulations focused on Caucasian populations infected with influenza type A. As part of the simulation settings, individual body weight (*BW*_*i*_; kg) was sampled from the observed distribution in this sub-population involved in clinical trials:

$${BW}_{i}=77.3∙e^{\eta_{BW}},with\;\eta_{BW}∼N(0,0.22^{2})$$

The PK exposure (AUC, ng.h/mL) was derived from the individual *BW*_*i*_ and the individual clearance parameter (*CL*_*i*_) from the PK model (not presented in this manuscript) with TVCL corresponding to the typical value of the clearance without accounting for the between-subject variability:

$${CL}_{i}=TVCL∙e^{\eta_{CL}},with\;TVCL=5.38∙{\left(\frac{{BW}_{i}}{64.8}\right)}^{1.04}∙1.77,and\;\eta_{CL}∼N(0,0.41^{2})$$

$${AUC}_{i}=\frac{DOSE}{{CL}_{i}}*1000$$

The dose was assumed to be 0 mg if the patient received placebo, 40 mg if *BW*_*i*_ was <80 kg, and 80 mg if *BW*_*i*_ was ≥80 kg. The individual VK parameters for which the PK–VK model estimated some between-subject variability (V0, beta, and delta) were generated using the following formulae:

$${V0}_{i}=V0+\eta_{V0},with\;\eta_{V0}∼N(0,0.278^{2})$$

$$\beta_{i}=\beta ∙e^{\eta_{\beta }},with\;\eta_{\beta }∼N(0,0.284^{2})$$

$$\delta_{i}=\delta ∙e^{\eta_{\delta }},with\;\eta_{\delta }∼N(0,0.647^{2})$$

The other parameters were fixed to the population value.

Subsequently, the individual viral titer profiles were generated as simulation outputs accounting for variability in patient characteristics (body weight, TOS derived from the distribution observed in clinical trials), PK (individual exposure, derived from body weight and clearance), and drug–disease variability (from the individual VK parameters).

### Epidemiological models

Semi-mechanistic epidemiological models were developed based on dose–response-type models that translated influenza viral shedding into infectiousness–time profiles, assuming a functional relationship between nasal viral shedding and infectiousness. The three models were used to produce infectiousness profiles and assumed that infectiousness is proportional to viral titer on a natural-scale, logarithmic-scale, or to a dose–response transform of viral titer (${1-e}^{\left(-\alpha *V[t]\right)}$), where α >0 is a parameter that encompasses both the infection probability of a single virion in an exponential dose–response model, as well as the shedding and transport efficiency of virus from the source infection. Higher values of α indicate that a person shedding some amount of virus has a higher probability of infecting another person at that point in time. Due to probability saturation effects, we observed empirically that values of α ~ 1e-3 approximate log-scale models, and due to the mathematical structure of the model very small values of α converge to natural-scale models. Thus, the models are mechanistically derived and approximately interpolate between common assumptions while also proposing new ones. For a more detailed description of the epidemiological models, please refer to S1 Text (Technical appendix).

### Population infectiousness and transmission mitigation

The epidemiological models were paired with the simulations from the within-host PK–VK model for viral shedding and were used to predict the reduction in influenza transmission (i.e., transmission mitigation) that would be expected after treatment with baloxavir or oseltamivir. This was accomplished by applying the appropriate transform function for each epidemiological model to the simulated viral shedding curve for each of the 1000 simulated individuals and then computing the area under the curve of the resulting infectiousness profile to consider transmissibility over the full course of infection. For each individual we computed the ratio of their total infectiousness when treated with an antiviral or when untreated (placebo). The complement of the infectiousness ratio value is the transmission mitigation. The effect of treatment timing after symptom onset was also explored by adjusting the individual simulations from the PK−VK model to reflect the timing and type of antiviral treatment. It was assumed that that for each individual, their infectiousness at any point in time is proportional to the infectiousness profile curve, and with a constant contact rate in time, an individual’s total infectiousness is the time-integrated area under that curve.

Model predictions of secondary case reduction with baloxavir were compared with recent clinical data from the Phase 3 BLOCKSTONE study, quantifying the impact of baloxavir as post-exposure prophylaxis in the household setting [14].

### BLOCKSTONE sub-analysis

Patients from the modified intention-to-treat population of BLOCKSTONE that had Reverse transcription polymerase chain reaction (RT-PCR)-confirmed influenza, fever (≥37.5C), and at least one respiratory symptom were included in the analysis. Patients with an event were sorted by time from influenza onset to treatment (0–<24 or ≥24–48 hours) and by treatment received. All analyses were performed using SAS version 9.4. Results are reported in S2 Table.

## Supporting information

S1 Table Overview of studies included in PK–VK model development. *T0821 (JapiCTI-153090) was a double-blind, dose-ranging, randomized trial (1:1:1:1) of single doses of baloxavir (10, 20, or 40 mg) versus placebo in OwH adult patients (age 20 to 64 years) with acute, uncomplicated influenza infection.[3] T0831 (NCT02954354; CAPSTONE-1) was a double-blind, randomized trial (2:2:1) of baloxavir versus oseltamivir or placebo in OwH adolescent and adult patients (age 12 to 64 years and ≥40 kg) with acute, uncomplicated influenza infection.[3] T0832 (NCT02949011; CAPSTONE-2) was a double-blind, randomized trial (1:1:1) of baloxavir versus oseltamivir or placebo in high-risk adolescent and adult patients (age ≥12 years and ≥40 kg) with acute, uncomplicated influenza infection.[5] T0821, T0831 and T0832 assessed the change from baseline in influenza virus titer over time.[3,5] In T0821, nasal or throat swabs were collected pre-dose at Visit 1 (Day 1), Visit 2 (Day 2), Visit 3 (Day 3; if circumstances permitted), Visit 4 (Days 5 to 7) and Visit 5 (Days 8 to 11).[25] In T0831 and T0832, nasopharyngeal/pharyngeal swabs were collected pre-dose on Day 1, and on study Days 2–6 and Day 9; Days 4 and 6 were optional visits.[3,5,25] ^†^Data are shown for the influenza-positive ITTI population in T0821 and T0831, and for the modified ITTI population in T0832. ^‡^75 mg oseltamivir twice a day for 5 days. OwH, otherwise healthy; ITTI, intention-to-treat infected.
(DOCX)

S2 Table Secondary cases of influenza infection observed in the Phase 3 BLOCKSTONE study following treatment of index patients. (DOCX)

S1 Fig Example individual viral shedding curves using observed data and estimated data from the PK−VK model. (TIF)

S2 Fig Goodness-of-fit plot between observed and estimated data for individuals using the PK−VK model. (TIF)

S3 Fig Population-level infectiousness over time. (DOCX)

S1 Text Technical appendix. (DOCX)

S1 Zip Folder Folder containing relevant data and codes. Contents include R files of programming scripts used to generate data, simulation data, and data used to generate figures in the manuscript.
(ZIP)
