# Supplementary material for: Novel modelling approaches to predict the role of antivirals in reducing influenza transmission
Source: PLoS Comput Biol. 2023 Jan 6;19(1):e1010797. doi: 10.1371/journal.pcbi.1010797 (PMC9876374; doi:10.1371/journal.pcbi.1010797)
Supplement: S1 Table — *T0821 (JapiCTI-153090) was a double-blind, dose-ranging, randomized trial (1:1:1:1) of single doses of baloxavir (10, 20, or 40 mg) versus placebo in OwH adult patients (age 20 to 64 years) with acute, uncomplicated influenza infection.[3] T0831 (NCT02954354; CAPSTONE-1) was a double-blind, randomized trial (2:2:1) of baloxavir versus oseltamivir or placebo in OwH adolescent and adult patients (age 12 to 64 years and ≥40 kg) with acute, uncomplicated influenza infection.[3] T0832 (NCT02949011; CAPSTONE-2) was a double-blind, randomized trial (1:1:1) of baloxavir versus oseltamivir or placebo in high-risk adolescent and adult patients (age ≥12 years and ≥40 kg) with acute, uncomplicated influenza infection.[5] T0821, T0831 and T0832 assessed the change from baseline in influenza virus titer over time.[3,5] In T0821, nasal or throat swabs were collected pre-dose at Visit 1 (Day 1), Visit 2 (Day 2), Visit 3 (Day 3; if circumstances permitted), Visit 4 (Days 5 to 7) and Visit 5 (Days 8 to 11).[25] In T0831 and T0832, nasopharyngeal/pharyngeal swabs were collected pre-dose on Day 1, and on study Days 2–6 and Day 9; Days 4 and 6 were optional visits.[3,5,25] †Data are shown for the influenza-positive ITTI population in T0821 and T0831, and for the modified ITTI population in T0832. ‡75 mg oseltamivir twice a day for 5 days. OwH, otherwise healthy; ITTI, intention-to-treat infected. (DOCX) [file pcbi.1010797.s001.docx]

**S1 Table Overview of studies included in PK–VK model development**

|  | **T0821  (JapiCTI-153090)*** | | | **T0831 (NCT02954354)*** | | **T0832 (NCT02949011)*** | |
| --- | --- | --- | --- | --- | --- | --- | --- |
| **Time from symptom onset to initiation of study treatment, n (%)**^†^[1, 2] | | | | | | | |
| **Placebo**  ≥0 to ≤12 hours  >12 to ≤24 hours  >24 to ≤36 hours  >36 to ≤48 hours | 11 (11.0)  42 (42.0)  22 (22.0)  25 (25.0) | | | 34 (14.7)  87 (37.7)  67 (29.0)  43 (18.6) | | 42 (11)  150 (39)  120 (31)  74 (19) | |
| **Baloxavir**  ≥0 to ≤12 hours  >12 to ≤24 hours  >24 to ≤36 hours  >36 to ≤48 hours | **10 mg**  7 (7.0)  38 (38.0)  30 (30.0)  25 (25.0) | **20 mg**  15 (15.0)  40 (40.0)  18 (18.0)  27 (27.0) | **40 mg**  12 (12.0)  28 (28.0)  36 (36.0)  24 (24.0) | 60 (13.2)  178 (39.0)  139 (30.5)  79 (17.3) | | 27 (7)  151 (39)  114 (29)  95 (24) | |
| **Oseltamivir**^‡^  ≥0 to ≤12 hours  >12 to ≤24 hours  >24 to ≤36 hours  >36 to ≤48 hours | –  –  –  – | | | 41 (10.9)  163 (43.2)  94 (24.9)  79 (21.0) | | 37 (10)  119 (31)  141 (36)  92 (24) | |
| **Population included in PK–VK model development, n** | | | | | | | |
| **Placebo** | 98 | | | 216 | | 356 | |
| **Baloxavir** | **10 mg**  99 | **20 mg**  100 | **40 mg**  97 | **40 mg**  362 | **80 mg**  73 | **40 mg**  223 | **80 mg**  131 |
| **Oseltamivir**^‡^ | – | | | 365 | | 358 | |

^*^T0821 (JapiCTI-153090) was a double-blind, dose-ranging, randomized trial (1:1:1:1) of single doses of baloxavir (10, 20, or 40 mg) versus placebo in OwH adult patients (age 20 to 64 years) with acute, uncomplicated influenza infection.[1] T0831 (NCT02954354; CAPSTONE-1) was a double-blind, randomized trial (2:2:1) of baloxavir versus oseltamivir or placebo in OwH adolescent and adult patients (age 12 to 64 years and ≥40 kg) with acute, uncomplicated influenza infection.[1] T0832 (NCT02949011; CAPSTONE-2) was a double-blind, randomized trial (1:1:1) of baloxavir versus oseltamivir or placebo in high-risk adolescent and adult patients (age ≥12 years and ≥40 kg) with acute, uncomplicated influenza infection.[2] T0821, T0831 and T0832 assessed the change from baseline in influenza virus titer over time.[1, 2] In T0821, nasal or throat swabs were collected pre-dose at Visit 1 (Day 1), Visit 2 (Day 2), Visit 3 (Day 3; if circumstances permitted), Visit 4 (Days 5 to 7) and Visit 5 (Days 8 to 11).[3] In T0831 and T0832, nasopharyngeal/pharyngeal swabs were collected pre-dose on Day 1, and on study Days 2–6 and Day 9; Days 4 and 6 were optional visits.[1-3]

^†^Data are shown for the influenza-positive ITTI population in T0821 and T0831, and for the modified ITTI population in T0832.

^‡^75 mg oseltamivir twice a day for 5 days

OwH, otherwise healthy; ITTI, intention-to-treat infected

**References**

1. Hayden FG, Sugaya N, Hirotsu N, Lee N, de Jong MD, Hurt AC, et al. Baloxavir Marboxil for Uncomplicated Influenza in Adults and Adolescents. N Engl J Med. 2018;379(10):913-23. Epub 2018/09/06. doi: 10.1056/NEJMoa1716197. PubMed PMID: 30184455.

2. Ison MG, Portsmouth S, Yoshida Y, Shishido T, Mitchener M, Tsuchiya K, et al. Early treatment with baloxavir marboxil in high-risk adolescent and adult outpatients with uncomplicated influenza (CAPSTONE-2): a randomised, placebo-controlled, phase 3 trial. Lancet Infect Dis. 2020;20(10):1204-14. Epub 2020/06/12. doi: 10.1016/S1473-3099(20)30004-9. PubMed PMID: 32526195.

3. FDA Review and Evaluation. NDA 210854. Xofluza (baloxavir marboxil) 2018 [cited 2022 12 October]. Available from: https://www.accessdata.fda.gov/drugsatfda_docs/nda/2018/210854Orig1s000TOC.cfm.
