# Supplementary material for: Novel modelling approaches to predict the role of antivirals in reducing influenza transmission
Source: PLoS Comput Biol. 2023 Jan 6;19(1):e1010797. doi: 10.1371/journal.pcbi.1010797 (PMC9876374; doi:10.1371/journal.pcbi.1010797)
Supplement: S2 Table — (DOCX) [file pcbi.1010797.s002.docx]

**S2 Table. Secondary cases of influenza infection observed in the Phase 3 BLOCKSTONE study following treatment of index patients.**

| **Timing of treatment initiation after symptom onset (index patient)** | **Index patient treatment** | **N** | **Number of secondary cases, n (%)** | **Percentage change** |
| --- | --- | --- | --- | --- |
| Overall | Baloxavir | 197 | 21 (10.7) | 36.7 |
| Overall | Other* | 178 | 30 (16.9) |  |
| 0–<24 hours | Baloxavir | 137 | 12 (8.8) | 46.3 |
| 0–<24 hours | Other* | 134 | 22 (16.4) |  |
| 24–48 hours | Baloxavir | 60 | 9 (15.0) | 17.6 |
| 24–48 hours | Other* | 44 | 8 (18.2) |  |

*Antiviral treatment other than baloxavir marboxil. In BLOCKSTONE 52.7% of index patients received baloxavir, 31.4% oseltamivir, and 16.0% another NAI [1].

References

1. Ikematsu H, Hayden FG, Kawaguchi K, Kinoshita M, de Jong MD, Lee N, et al. Baloxavir Marboxil for Prophylaxis against Influenza in Household Contacts. N Engl J Med. 2020;383(4):309-20. Epub 2020/07/09. doi: 10.1056/NEJMoa1915341. PubMed PMID: 32640124.
