# Supplementary figures and images for: Novel modelling approaches to predict the role of antivirals in reducing influenza transmission
Source: PLoS Comput Biol. 2023 Jan 6;19(1):e1010797. doi: 10.1371/journal.pcbi.1010797 (PMC9876374; doi:10.1371/journal.pcbi.1010797)

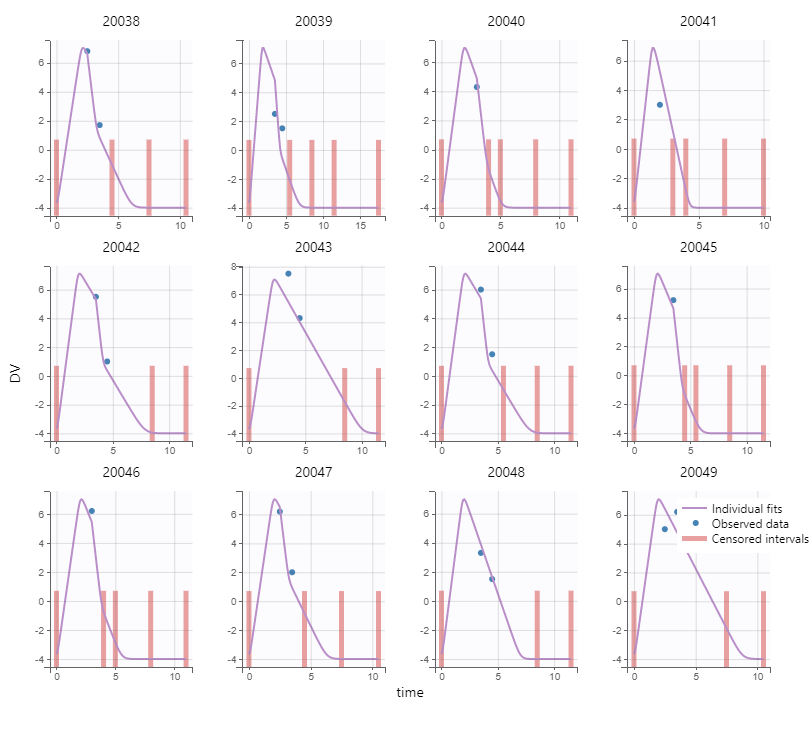

Supplement: S1 Fig — (TIF) [file pcbi.1010797.s003.tif]

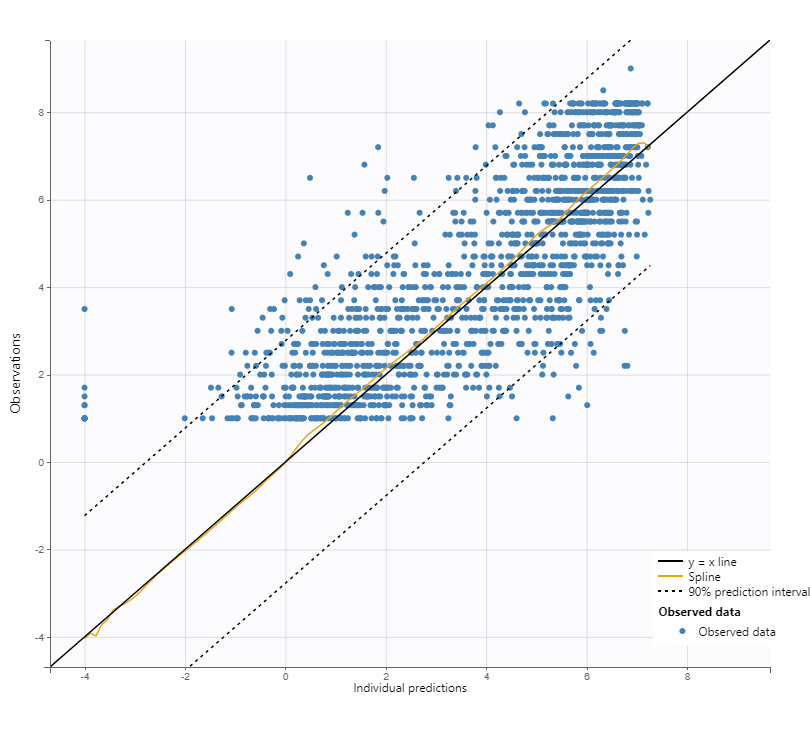

Supplement: S2 Fig — (TIF) [file pcbi.1010797.s004.tif]

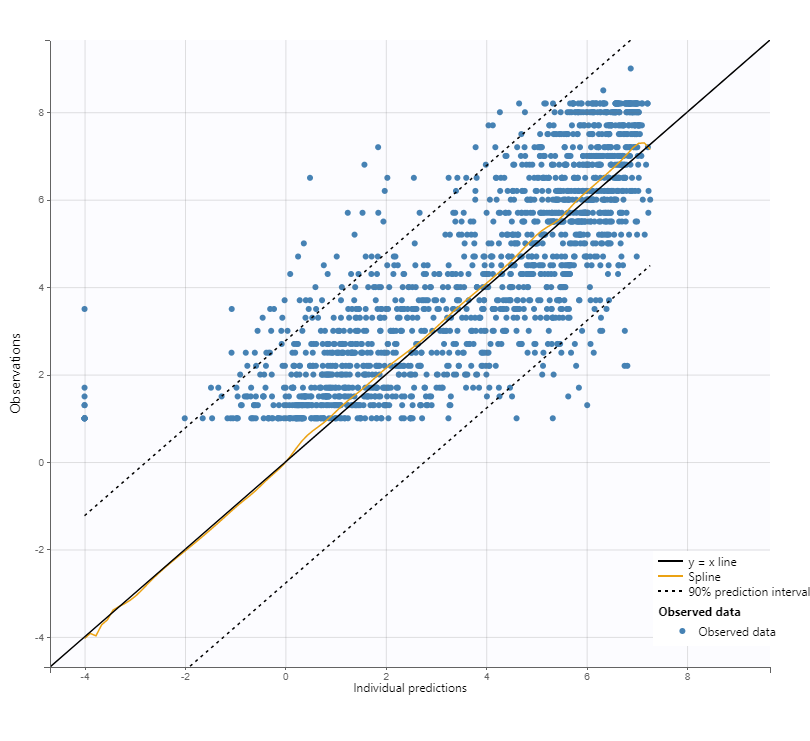

Supplement: S1 Zip Folder — Contents include R files of programming scripts used to generate data, simulation data, and data used to generate figures in the manuscript. (ZIP) [file pcbi.1010797.s007.zip › Data and model codes/goodness of fit plot.png]

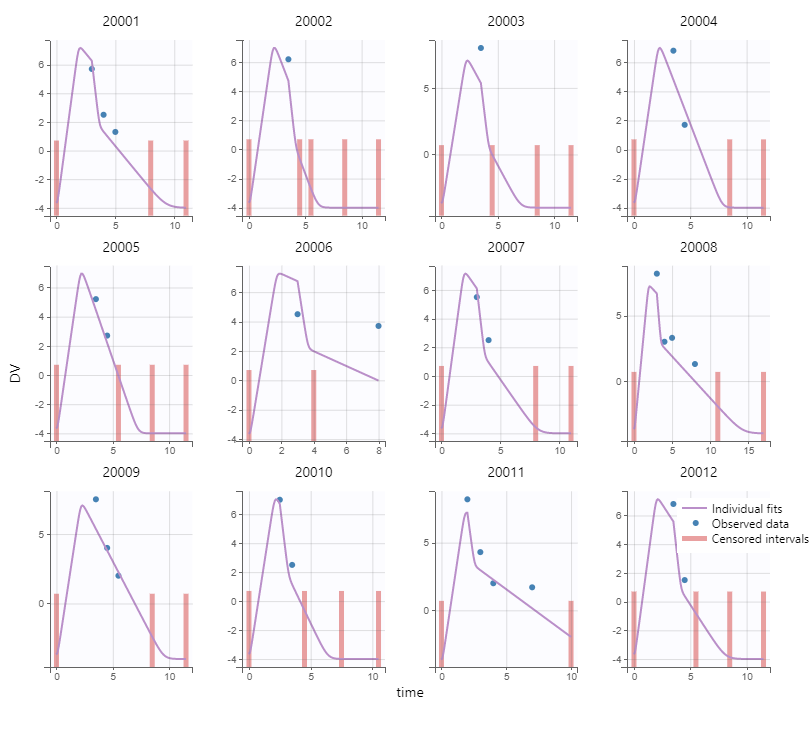

Supplement: S1 Zip Folder — Contents include R files of programming scripts used to generate data, simulation data, and data used to generate figures in the manuscript. (ZIP) [file pcbi.1010797.s007.zip › Data and model codes/indfits_DV_0_0.png]

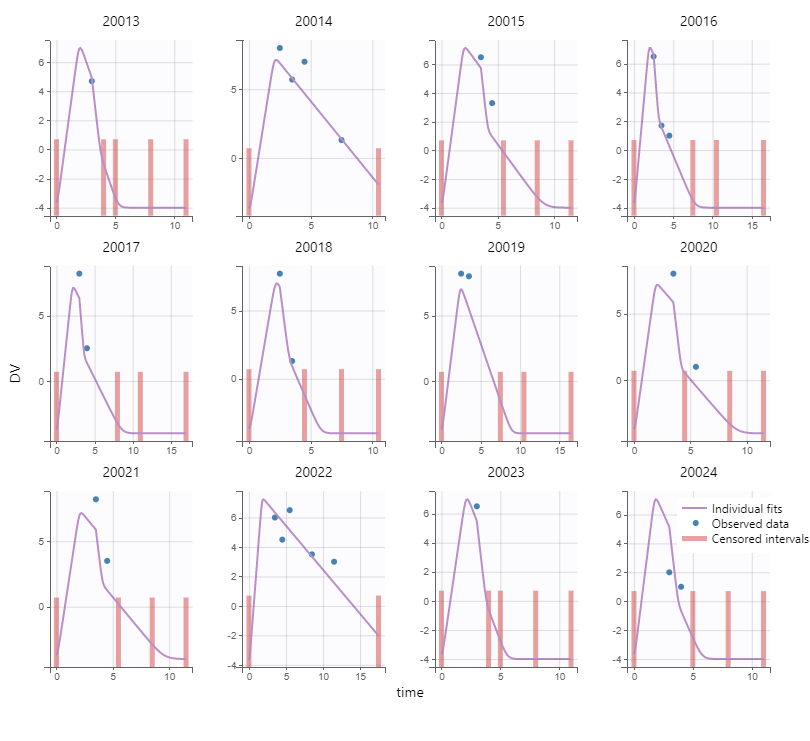

Supplement: S1 Zip Folder — Contents include R files of programming scripts used to generate data, simulation data, and data used to generate figures in the manuscript. (ZIP) [file pcbi.1010797.s007.zip › Data and model codes/indfits_DV_0_1.png]

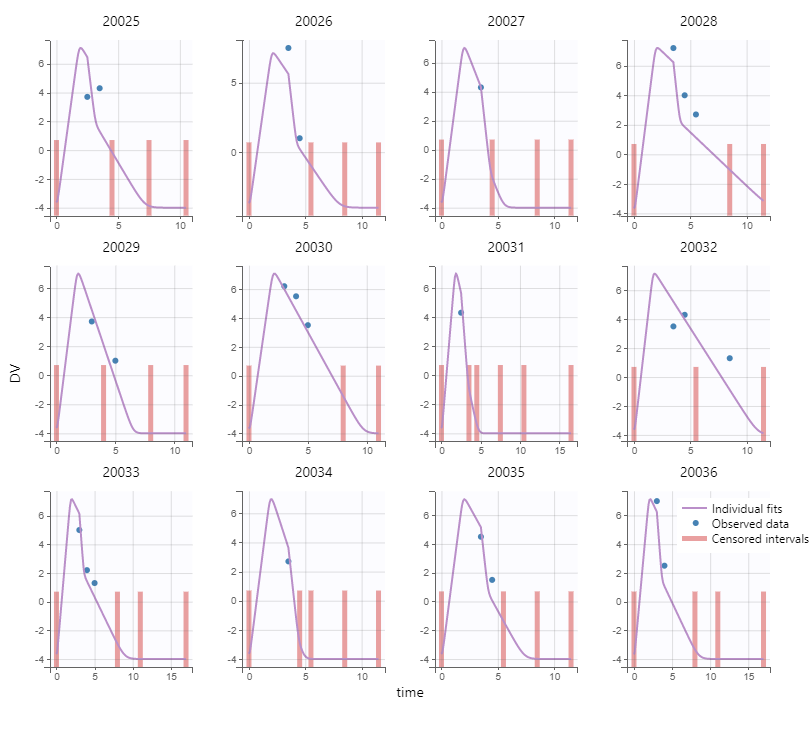

Supplement: S1 Zip Folder — Contents include R files of programming scripts used to generate data, simulation data, and data used to generate figures in the manuscript. (ZIP) [file pcbi.1010797.s007.zip › Data and model codes/indfits_DV_0_2.png]

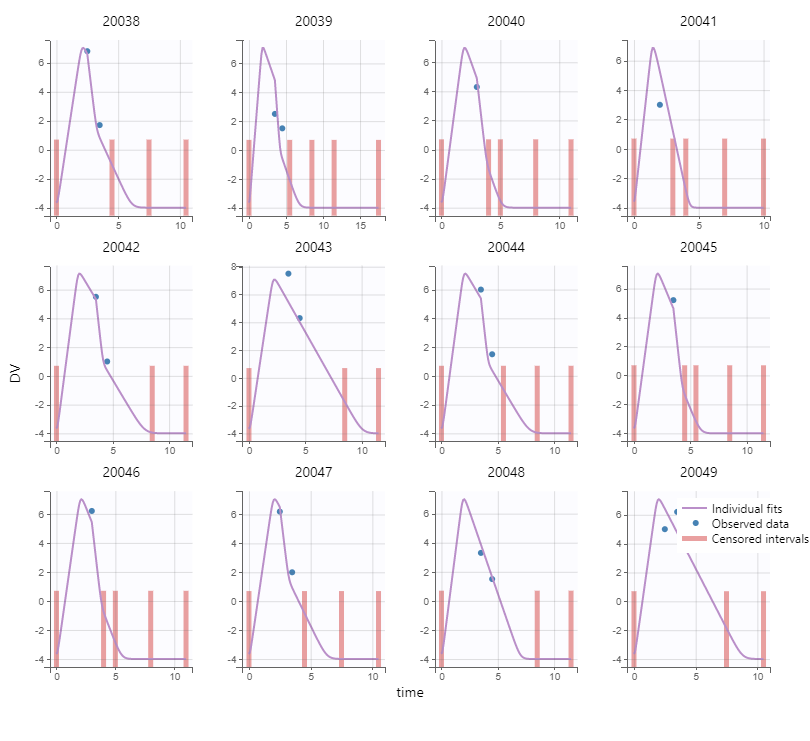

Supplement: S1 Zip Folder — Contents include R files of programming scripts used to generate data, simulation data, and data used to generate figures in the manuscript. (ZIP) [file pcbi.1010797.s007.zip › Data and model codes/indfits_DV_0_3.png]
