# Supplementary material for: Novel modelling approaches to predict the role of antivirals in reducing influenza transmission
Source: PLoS Comput Biol. 2023 Jan 6;19(1):e1010797. doi: 10.1371/journal.pcbi.1010797 (PMC9876374; doi:10.1371/journal.pcbi.1010797)
Supplement: S3 Fig — (DOCX) [file pcbi.1010797.s005.docx]

**S3 Fig. Population-level infectiousness over time**


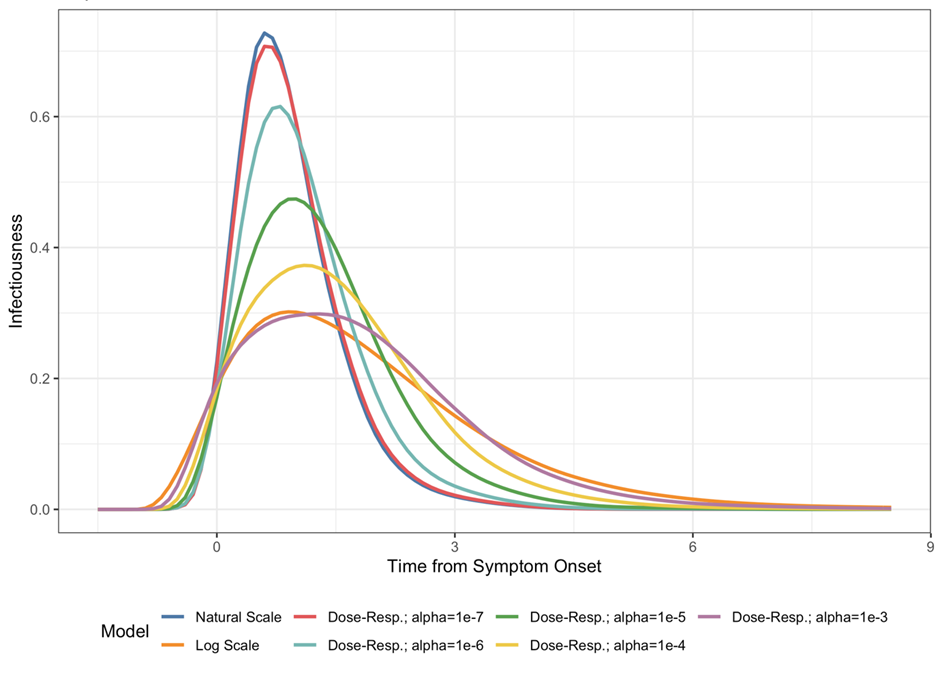


This approach can be generalised to consider greater heterogeneity in emission and transport, infectiousness, and contact. One natural extension of this model is the beta-Poisson dose–response approximation, which may better capture influenza infectiousness. However, this would be at the cost of additional parameters that would need to be estimated. Here, we have used the simplest one-parameter model for the sake of parsimony.
