# Supplementary material for: Novel modelling approaches to predict the role of antivirals in reducing influenza transmission
Source: PLoS Comput Biol. 2023 Jan 6;19(1):e1010797. doi: 10.1371/journal.pcbi.1010797 (PMC9876374; doi:10.1371/journal.pcbi.1010797)
Supplement: S1 Text — (DOCX) [file pcbi.1010797.s006.docx]

**S1 Text. Technical appendix**

*Derivation of dose–response epidemiological model*

$V\left( t \right)$ was the viral load at time t, determined by the pharmacokinetic–pharmacodynamic model for an infected person. For the dose–response model, it was assumed that a suitably close contact of that person at time t was exposed to a Poisson-distributed number of infectious virions, with a mean proportional to $V\left( t \right).$Therefore, if $D(t)$ was this dose, then:

$$D\left( t \right)\sim_{d}Pois(\beta V\left( t \right))$$

where $\beta>0$ is a constant that encodes the efficiency of viral emission and transport from the source to the contact.

It was assumed that infection in the contact is exponential, so that each virion in the absorbed dose has some independent probability $p$ of leading to an infection. The probability of infection $I\left( t \right)$ in this contact at time t was then:

$$I\left( t \right)=1-{(1-p)}^{D\left( t \right)}$$

The expected probability of infection was then:

$$\mathbb{E}I\left( t \right)=1- \sum_{n=0}^{\infty} \frac{\left( 1-p \right)^{n}{\beta V\left( t \right)}^{n}e^{-\beta V\left( t \right)}}{n!}=1- \sum_{n=0}^{\infty} \frac{{\left( (1-p \right)\beta V\left( t \right))}^{n}e^{-\beta V\left( t \right)}}{n!}= 1- \sum_{n=0}^{\infty} \frac{{\left( (1-p \right)\beta V\left( t \right))}^{n}e^{-\beta V\left( t \right)}}{n!}= 1-e^{\left( 1-p \right)\beta V\left( t \right)} e^{-\beta V\left( t \right)}= 1- e^{-p\beta V\left( t \right)}$$

If $\alpha=p \beta>0$ , the final form was obtained, where the expected probability of infection was:

$$1- e^{-\alpha V\left( t \right)}$$

whereby $\alpha$ encapsulated the efficiency of emission and transport in units that are relative to the infectiousness of a single virion. Here, the infection of a suitably close contact was modelled as a simple Bernoulli process; therefore, there was no difference between the expected probability of infection derived from the hierarchical model of dose and response, and the probability of infection derived from the Bernoulli model.

The other models that we considered in this study were the natural-scale model, where individual infectiousness was assumed to be proportional to $V\left( t \right)$, and the log-scale model where individual infectiousness was to be proportional to $log10 \left( V\left( t \right) \right)$ and constrained to be non-negative, i.e. to where $V\left( t \right)\geq1$. For all models, infectiousness was normalized so the average person would have unit area under their infectiousness curve. When antiviral treatment was considered, the reduction in secondary transmission for individuals was computed by comparing the area under the respective model-transformed infectiousness profiles. The PK-VK model allowed us to consider both the expected variation in the population of these infectiousness profiles as well as the varying impact of the introduction of antiviral treatment at different points in time.

Note that if $\alpha$ was sufficiently small, the dose–response model approximated the natural-scale model as $e^{-\alpha V\left( t \right)} \approx1- \alpha V\left( t \right)$, and thus infectiousness was directly proportional to $V\left( t \right)$. Empirically, the dose–response model produced similar population-level average infectiousness–time profiles to the log-scale model for values of $\alpha$ of around ${10}^{-3}$ due to partial probability saturation (S2 Fig). Therefore, the dose–response model interpolated between commonly used infectiousness assumptions while also proposing new ones.

**Fig. Population-level infectiousness over time**


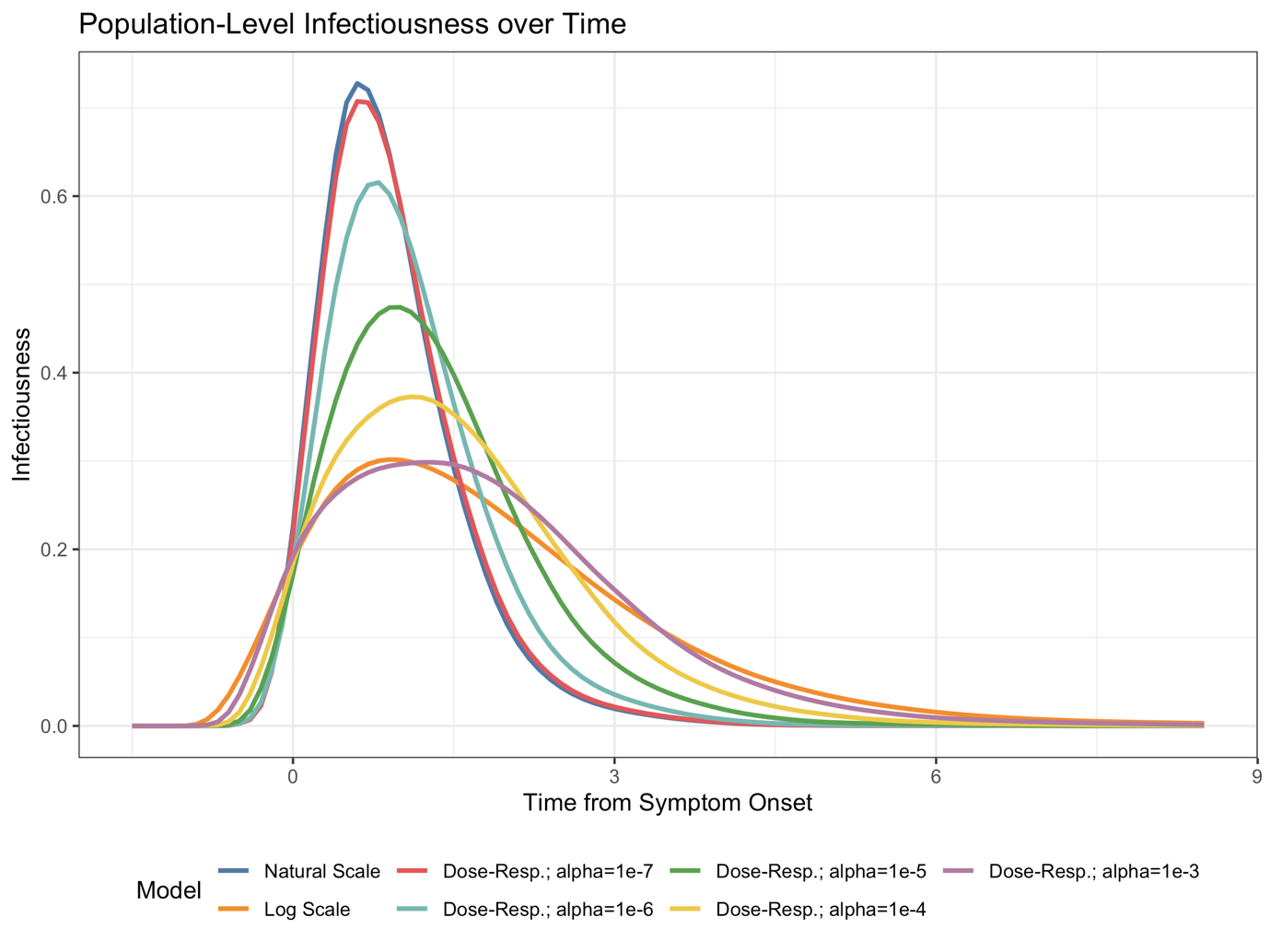


This approach can be generalised to consider greater heterogeneity in emission and transport, infectiousness, and contact. One natural extension of this model is the beta-Poisson dose–response approximation, which may better capture influenza infectiousness. However, this would be at the cost of additional parameters that would need to be estimated. Here, we have used the simplest one-parameter model for the sake of parsimony.
